# Damage Control Orthopedics Management as Vital Procedure in Elderly Patients with Femoral Neck Fractures Complicated with Chronic Renal Failure: A Retrospective Cohort Study

**DOI:** 10.1371/journal.pone.0154906

**Published:** 2016-05-05

**Authors:** Chenhui Dong, Yunjiao Wang, Ziming Wang, Yu Wang, Siyu Wu, Quanyin Du, Aimin Wang

**Affiliations:** Department of Orthopedics, Institute of Surgery Research, Daping Hospital, Third Military Medical University, Chongqing, 400042, China; Mayo Clinic Minnesota, UNITED STATES

## Abstract

**Background:**

Chronic renal failure (CRF) predisposes to hip fractures in elderly patients, with high subsequent mortality. Selection and timing of the surgical procedure of such patients is a serious challenge. Many clinicians believe in earlier surgery as preferable and providing better outcomes. Damage control orthopedics (DCO) aids to adjust and optimize the overall condition of patients.

**Methods:**

In 32 patients with femoral neck fractures complicated with CRF, we evaluated how the timing of the surgery determines the mortality rates if the DCO approach is applied. Preoperative ASA grading, POSSUM score, P-POSSUM score and DCO were carried out. Based on the assessment, timing of the surgery was ascertained.

**Results:**

Of a total of 32 patients, twenty-nine patients were accepted for either early (< 48 hours; n = 18) or delayed (3–10 days; n = 10) surgery. Hip arthroplasty (total hip arthroplasty and hemiarthroplasty) was the principal surgery option. All patients survived operation and were followed up postoperatively with the average time of 30 days. Postoperative complications tended to occur at higher rates in the early vs. delayed surgery group (7/18 vs. 5/10). During follow up, a total of 3 patients died in both groups (2/18 in the early surgery and 1/10 in the delayed surgery group), mostly from multi-organ failures and acute respiratory distress syndrome. There was no significant difference in complication rates and Harris hip score between both groups.

**Conclusion:**

In patients with femoral neck fracture complicated with CRF, delaying the surgery for several days does not increase the incidence of postoperative adverse events.

## Introduction

Frailty and osteoporosis predispose the elderly patients to minor traumatic falls and hip fractures [1]. These fractures are especially frequent in patients with concomitant chronic organ failure [2–4], such as chronic renal failure, a chronic kidney disease (CKD) with diminished glomerular filtration rate. The decompensated period of chronic renal failure is accompanied by electrolyte and metabolic disorders, and multiple organ system dysfunctions [4]. Renal-related osteodystrophy predisposes to osteoporosis, osteomalacia and spontaneous fractures [5, 6]. Most hip fractures associated with chronic renal failure occur in the elderly. About one-third of these patients die within a year after the surgery, and about 75% of the deaths is related to comorbidities [7, 8].

The damage control theory was first proposed by Rotondo in 1993 [9] and was later applied to patients with serious multiple trauma. The objective of damage control orthopedics (DCO) is to avoid deterioration of patient’s clinical condition caused by the “second hit” of the surgical procedure. This is achieved by adjusting and optimizing the overall condition of patients, and, ultimately, selecting the appropriate timing of the surgery [10].

When patients with serious organ failures undergo trauma, systemic inflammatory responses response is instigated [11]. This response negatively affects the patient prognosis because of coagulation disorders, fluid and electrolyte disturbances, hypothermia, acidosis and shock. Therefore, there was no standardized treatment for elderly patients with femoral neck fracture accompanied with the end-stage CKD, and potential beneficial effect of DCO on patient prognosis were not well understood [12, 13]. Selection and timing of the surgical procedure and perioperative management of patients suffering from femoral neck fracture complicated with chronic renal failure poses a serious challenge to the surgeons.

In this study, we treated 32 patients with femoral neck fractures complicated with chronic renal failure and evaluated how the timing of the surgery determines the mortality rates if DCO approach is applied.

## Materials and Methods

### Patients

This study was approved by the Ethics Committee of Daping Hospital, Third Military Medical University. Each participants in the study provided written informed consent. All patients had femoral neck fractures and concomitant chronic renal failure. The study enrolment proceeded from January 2008 through December 2014. The inclusion criteria were the following: (i) hip fracture, (ii) chronic renal failure and renal dysfunction, (iii) complete medical records. The exclusion criteria were the following: (i) multiple (injury) fracture or open hip fracture, and (ii) incomplete medical records. Thirty-two patients were selected according to inclusion criteria. Thirty-two patients were selected according to inclusion criteria. Patient data were obtained in the Electronic Medical Records system of the Daping Hospital and Institute of Field Surgery. The surgery outcome was established by the outpatient follow-up.

### Surgical risk evaluation integrating DCO

Renal function was ascertained according to the CKD staging criteria [14]. Number, category and severity of underlying diseases were ascertained according to patient history, and physical and laboratory examinations. Furthermore, ASA grading, POSSUM and P-POSSUM score were utilized to assess the likelihood of postoperative complications (both the incidence and the risk of mortality thereof).

### Patient recuperation

Patients in the CKD4 or higher stage underwent preparatory treatment before dialysis. Patients in exacerbation received hemodialysis therapy. Dialysis indications for patients with diabetic nephropathy can be relaxed [15]. All patients with dialysis indications underwent preoperative dialysis. An important criterion was that blood pressure before the first dialysis should be less than 130/80 mm Hg.

Apart from the CKD treatment, other coexistent underlying diseases and metabolic disorders were also actively treated.

### Selection of optimal time for surgery and anesthesia

We determined the surgery timing based on the following considerations. Early surgery was considered feasible in patients in good general condition and assessed preoperatively as at “low risk”. In contrast, the surgery was to be delayed in patients with multiple underlying diseases, and those in poor general condition and assessed to be at “high risk”. The latter patients were recommended to continue receiving recuperating treatment in order to increase the surgery tolerance.

Specifically, based on published evidence [16–20] and own experience, the pre-surgery evaluation was set as follows. If patient’s risk assessment yielded ASA grading of < III, POSSUM predicted morbidity rate of < 60%, and P-POSSUM predicted mortality rate of < 20%, then perioperative physical condition of the patient was assumed as sufficiently well, and early (i.e., within 48 hours) surgery was considered. If, however, the risk assessment yielded the levels higher than those above, the patients were thoroughly nursed and recuperated for 1 to 3 days. Following this, the patients underwent the second risk assessment. If the risk assessment fell under the aforementioned criteria (ASA < III, POSSUM < 60%, and P-POSSUM < 20%), the surgery was to be performed immediately. But if the risk was still higher than these criteria, the patients were to be nursed and recuperated for another 1 to 3 days. After the third risk assessment, the decision was made as to whether to continue with the surgical treatment or use conservative treatment instead.

Thereby, treatment and evaluation were implemented until the surgical risk was deemed acceptable. Furthermore, anesthesia methods were personalized and selected according to the physiological index of patients.

### Surgical procedure

We selected the hip arthroplasty (i.e., total hip arthroplasty and hemiarthroplasty) as the principal surgery option. The rationale for this choice was the following. Our patients were senile, aged more than 60 years old, and mostly presented with hip osteoarthritis. Furthermore, they all had varying degrees of chronic renal failure. With the background of coexisting diseases, internal fixation might have led to complications (e.g., bone ununion, pressure sores, worsening of renal function after the surgery). In addition, we wanted to avoid having to remove the internal fixation plate. In patients with chronic renal failure, the second surgical procedure for removal the internal fixation plate might have been extremely dangerous, both because of surgery and anesthesia.

### Postoperative dialysis treatment

Patients with preoperative dialysis indications were sent to the ICU after the surgery for monitoring and dialysis. Early and continuous dialysis treatment was implemented according to hemoglobin, albumin, electrolytes, urine output and nephrology statuses after the surgery. If patients had concomitant diabetes, dialysis could be advanced. The dialysis channel was protected to prevent local hematoma and infection.

### Postoperative general treatment

Patients who did not require dialysis could still be sent to the ICU for monitoring and potential treatments based on their intraoperative condition. Renal function and electrolytes were monitored, and fluid volume was strictly controlled after the surgery to prevent water and electrolyte disturbance. The patients were treated with regular analgesia, and ECG and other vital signs were monitored. The component transfusion could be done if required, according to the condition of patients.

### Postoperative functional training

Individualized rehabilitation exercise program was carried out after the surgery. Generally, active hip flexion and extension movement were practiced 3–5 days after the surgery but not too vigorously to prevent dislocation. The patients began to stand beside the bed with protection and gradually practice walking with a rollator walker (or walking frame) 5–7 days after the surgery. Squatting was practiced with assistance starting from 3 months after the surgery. Furthermore, patients were instructed to be attentive and to prevent falls.

### Efficacy assessment

Postoperative surgical efficacy of patients was evaluated as follows. The parameters included operative time, blood loss, hospital stay, perioperative complications, and mortality. Regular follow-up was implemented after discharge. The hip joint function was assessed using the Harris hip score, with the score of 90–100 assessed as “superior”, 80–90 as “good”, 70–79 as “tolerable”, and < 70 as “poor”.

### Statistical analysis

The SPSS version 19.0 (Chicago, IL, USA) was used for statistical analysis. Qualitative data were presented as rates and compared using either the chi-square or the Fisher exact test. Quantitative data were presented as mean ± SD and compared using the *t* test. The Kaplan-Meier curve analysis was used to compare survival rates in patients with different surgical timing. The factors with the *p* < 0.05 were selected for the multivariate logistic regression analysis. Statistical significances were considered at the *p* < 0.05.

## Results

We included 32 patients with hip fracture and concomitant chronic renal failure (Table 1).

**Table 1:** Demographic and clinical characteristics of study patients.

Characteristics	Data
Total number of patients	32
Age (years; mean ± SD)	72.38 ± 7.16
Female patients, number	15
Causes of injuries	
Slight fall, number	27
Pathologic fracture, number	5
CKD staging	
Stage 2, number	4
Stage 3, number	2
Stage 4, number	11
Stage 5, number	15
History of dialysis treatment	
Dialysis treatment applied, number	27
Without dialysis, number	5

Footnote: CKD, chronic kidney disease.

As described in the Materials and Methods, the patients underwent 1 to 3 risk assessments. Thereby, the number of patients who could tolerate the surgery reached 28. The other 4 patients had continuing signs of deterioration. Therefore, it was decided to replace the surgery with non-surgical symptomatic and supportive treatment due to uncontrollable surgical risk. All 4 patients died within 30 days.

As repeated risk assessment revealed, the surgical risk of patients who needed continuing recuperation was significantly higher than that of patients that could undergo the surgery without delays (*p* < 0.05, Table 2).

**Table 2:** Preoperative risk assessments.

Assessment	Number of patients	POSSUM complication rate (%)	P-POSSUM mortality rate (%)
First assessment	32		
Ready for surgery	8	51.01 ± 6.72**	11.61 ± 2.86*
Continued to recuperate	24	78.07 ± 10.98	35.36 ± 16.57
Second assessment	24		
Ready for surgery	17	58.2 ± 4.19**	17.04 ± 1.73**
Continued to recuperate	7	83.65 ± 6.76	48.84 ± 12.87
Third assessment	7		
Ready for surgery	3	61.77 ± 1.59*	18.73 ± 1.37**
Conservative treatment	4	93.78 ± 1.91	63.59 ± 8.34

Footnote: Data are presented as mean ± SD.

* *p* < 0.05;

** *p* < 0.01.

The comparisons were between patients who underwent the first assessment *vs* patients who underwent the second assessment, and patients who underwent the third assessment *vs* patients who received non-surgical treatment.

Among 28 patients who underwent the surgery, there were 18 patients in whom the surgery was done within 48 hours. These patients comprised the early surgery group. Another 10 patients received the surgery in 3 to 10 days, and these patients comprised the delayed surgery group. There was no significant difference in age and gender between these two groups (Table 3).

**Table 3:** First preoperative risk assessments.

	Early surgery group	Delayed surgery group	*p*
Age (years, mean ± SD)	71.24± 6.52	73.8 ± 7.31	0.186
Female patients, number	10	5	0.174
Femoral neck fracture classification (number)			
Intra-capsular	15	8	0.875
Extra-capsular	3	2	0.886
CKD staging (mean ± SD)	3.67 ± 1.05	4.8 ± 0.4	0.002
Number of concomitant chronic diseases (mean ± SD)	4.82 ± 1.11	6.4± 1.96	0.038
POSSUM complication rate (%, mean ± SD)	62.09 ± 11.18	77.51 ± 8.85	0.001
P-POSSUM mortality rate (%, mean ± SD)	20.01 ± 9.03	30.26 ± 4.06	0.001
ASA grading, number			
grade I & II	5	2	0.768
grade III & IV	13	8	0.41

Footnote: CKD, chronic kidney disease

With regard to the femoral neck fracture classification, most patients in both groups had intra-capsular fractures. The number of concomitant diseases did not differ between groups. With regard to CKD staging, patient condition in the delayed surgery group was more severe than in the early surgery group (*p* < 0.05, Table 3). The surgical risk at the first assessment was also significantly higher in the former group (*p* = 0.002, Table 3). However, there was no significant difference in the ASA grading (Table 3).

**Fig 1:**
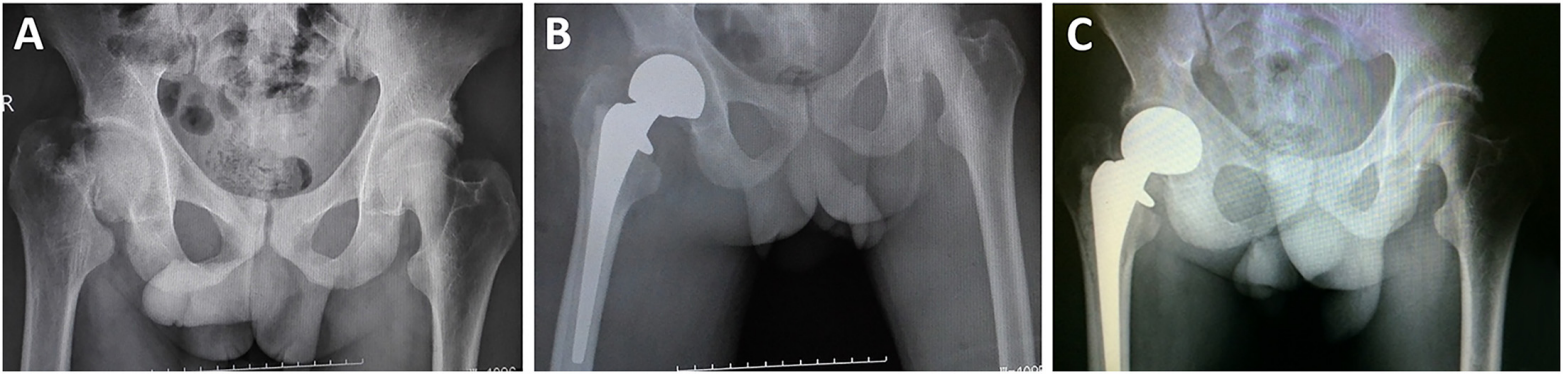
X-ray images of the right femoral neck fracture in a 72-year old male patient with chronic renal failure before and after hemiarthroplasty. (A) Preoperative imaging shows compression fractures on the femoral neck of the right hip and shortening deformity of the femoral neck. (B) Representative images taken 6 months after right femoral head arthroplasty. (C) At 24 months of follow-up, the prosthesis was in the correct position. On subsequent X-ray images, there were no further changes.

**Fig 2:**
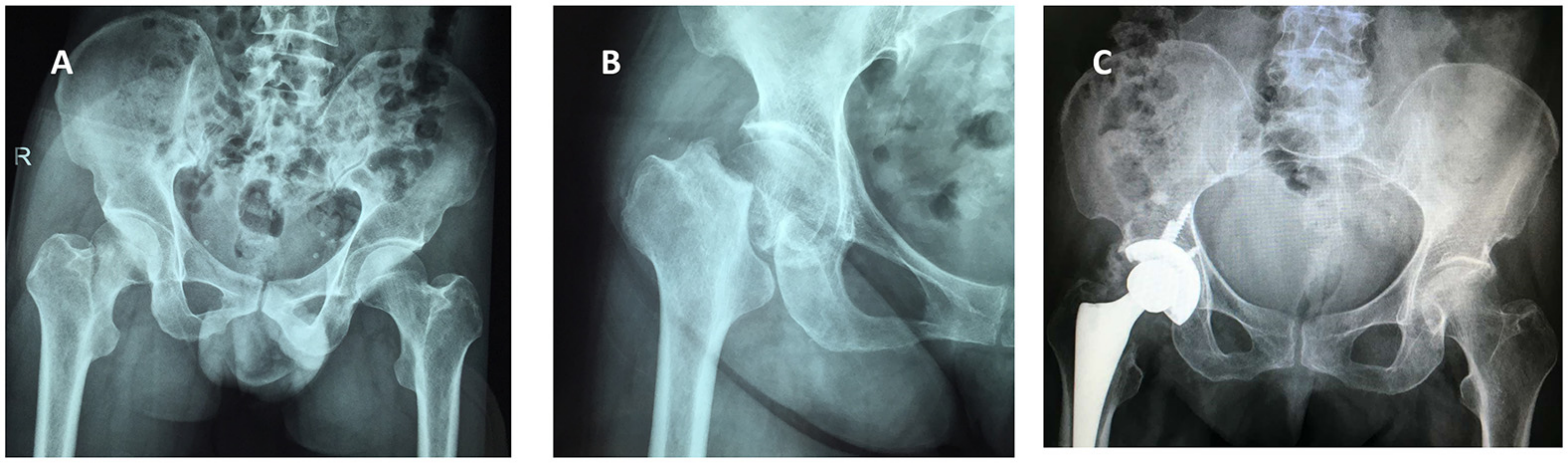
X-ray images of the right femoral neck fracture in a 65-year-old male patient with chronic renal failure before and after total hip arthroplasty. (A) Preoperative imaging shows compression fractures on the femoral neck of the right hip. (B) Representative images of the right femoral head fracture in lateral projection. (C) Representative images taken 3 days after total hip arthroplasty.

The utilized surgical approaches were hemiarthroplasty (Fig 1) and total hip arthroplasty (Fig 2). The anesthesia approach was mainly nerve block anesthesia, used to a comparable extent in both groups. Furthermore, both groups were comparable with regard to operative time, intraoperative bleeding volume, and hospitalization time. The hospitalization time, the operative time and the intraoperative bleeding volume were no significantly difference between those two groups (Table 4).

**Table 4:** Surgery parameters.

	Early surgery group	Delayed surgery group	*p*
Surgery procedures (number)	18	10	
Femoral head arthroplasty	11	9	0.986
Total hip arthroplasty	7	1	0.976
Bleeding volume (ml, mean ± SD)	463.89 ± 270.7	435 ± 251.05	0.396
Type of anesthesia (number)			
General	3	2	0.381
Spinal	3	2	0.084
Nerve block	12	6	0.093
Length of surgery (min, mean ± SD)	104.45 ± 11.65	110.5 ± 14.91	0.131
Length of hospital stays (days, mean ± SD)	20.83 ± 5.08	22.3 ± 5.66	0.252
Harris hip score (last follow-up)	89.06± 5.12	87.7 ± 5.18	0.262

In both groups combined, 12 patients (37.5%) presented with postoperative morbidity. Specifically, in the early surgery group complications were in observed in 7/18 patients: 1 patients with gastrointestinal hemorrhages, 3 patients with respiratory failure, 2 patients with cardiovascular complication, and 1 patients with postoperatively congnitive deficit. In the delayed surgery group, there were complications in 5/10 patients: 1 patient with respiratory complication, 1 patient with arrhythmia, 1 patient with postoperatively congnitive deficit, and 2 patients with gastrointestinal hemorrhages.

Within 30 days of follow up, 2/18 patients (11.12%) in the early surgery group died of serious morbidity. Specifically, one patient died of multi-organ failures and 1 of acute respiratory distress syndrome. The delayed surgery group had two deaths (1/10; 10%) of multi-organs failures.

**Fig 3:**
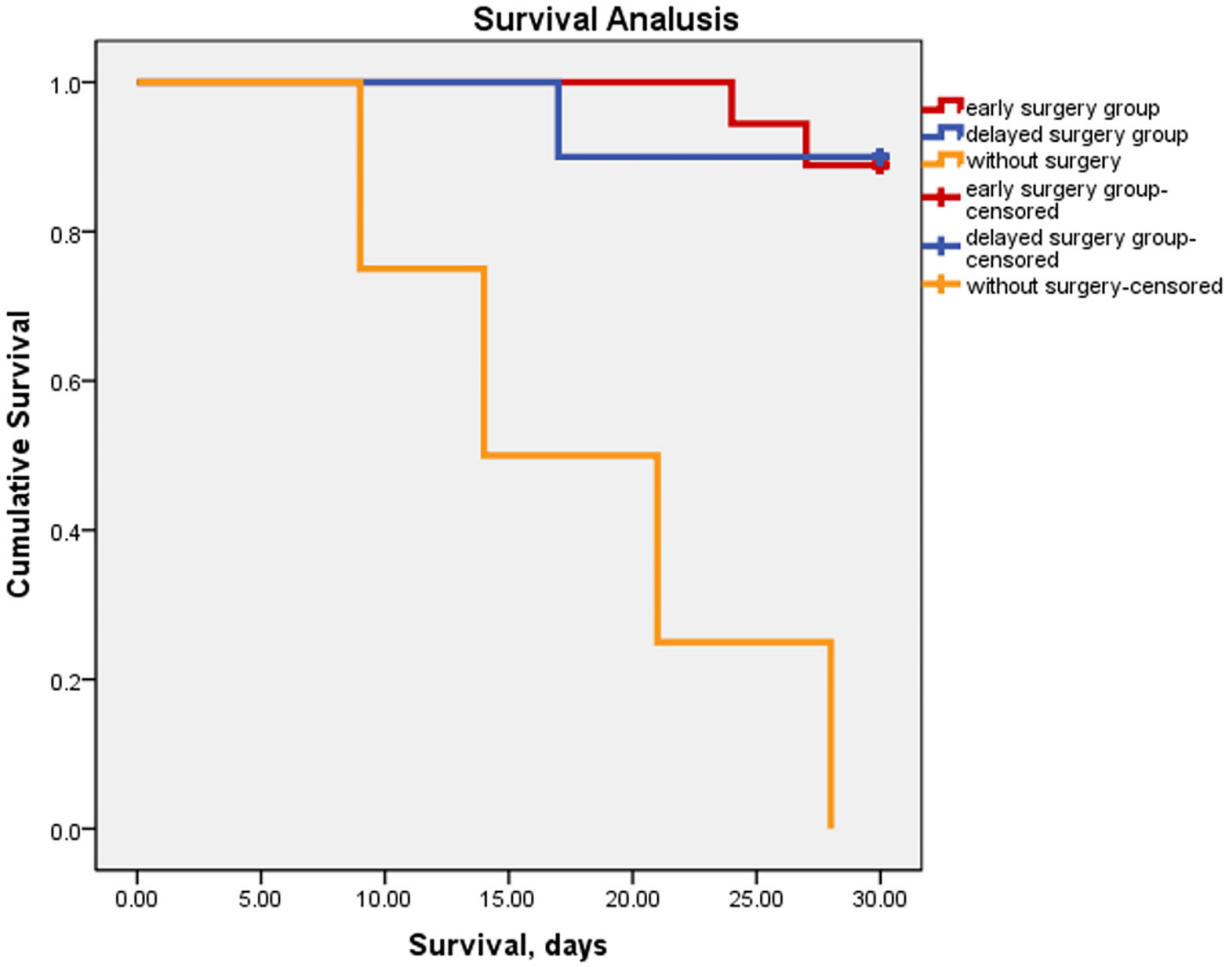
The Kaplan-Meier survival curves analysis in patients with or without surgical treatment. Survival curves in 32 study patients. Based on surgical risk assessment, 28 patients were selected for surgical treatment. The remaining 4 patients could not undergo the surgery and dead within 30 days. Furthermore, mortality rates in the early surgery group were equal with the delayed surgery group.

There was no significant difference in complication rates and Harris hip score between both groups in the last follow up. The survival rates (Kaplan-Meier survival curves) in patients with or without surgical treatment are shown in Fig 3. Interestingly, postoperative morbidity and mortality rates in all patients were significantly lower than the rates predicted preoperatively by POSSUM and P-POSSUM scoring (*p* < 0.01).

## Discussion

In this study, using DCO management, we evaluated how the timing of the surgery determines the mortality rates in patients with femoral neck fractures complicated with chronic renal failure.

Based on laboratory examinations, combined POSSUM and P-POSSUM scores, and ASA classification, detailed preoperative risk assessment was conducted. Underlying diseases and physiological disorders were addressed. Individual operation program development was carried out simultaneously with the step-by-step assessment. Surgical timing was determined based on assessment results. Patients suffering from CKD at the Stage 2 and 3 were treated with conventional perioperative management. Patients suffering from CKD at the Stage 4 and above were treated with dialysis prior to and postoperatively to reduce the risk of perioperative complications.

In the past, earlier surgery was considered as preferable and providing better outcomes. This was because of reports showing that delaying the surgery by more than 48 hours in the elderly hip fracture patients led to increase in post-surgery mortality rates [21]. Thus, Uzoigwe et al. [22] reported that delaying surgery increased mortality in hospitalized patients, whereas mortality of patients treated with early surgery was less. However, this was disputed by Leung et al. [23] who did not confirm that early surgery reduces mortality rates. Others even found that early surgery may increase postoperative mortality in patients in adverse physical condition [24]. Postponing surgery has a minor impact on postoperative mortality, but may increase the incidence of postoperative morbidity and complication [25, 26]. It is thus important to find a balance between patient’s physical condition and surgery timing. This requires a comprehensive preoperative risk assessment, damage control surgery and an experienced clinical team [27, 28].

Importantly, the DCO approach diminishes the complication and mortality rates to the rates significantly lower than preoperatively expected. Furthermore, using the DCO approach, delaying the surgery to up to 10 days did not adversely affect the outcome.

**Fig 4:**
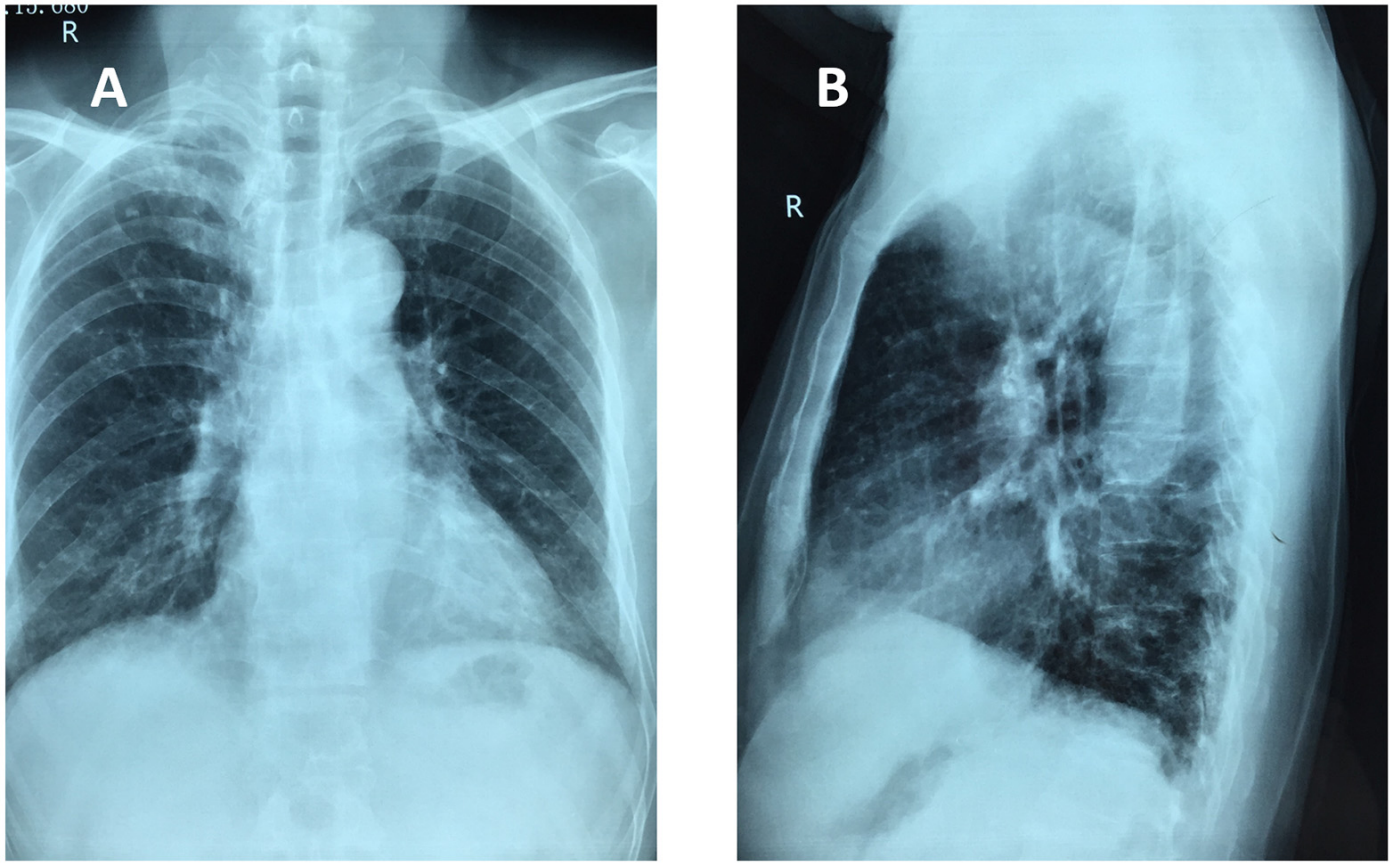
Chest X-ray images of a 72-year old male patient with femoral neck fracture complicated with chronic renal failure. (A) Frontal projection. (B) Lateral projection.

In our patients, we mostly used continuous epidural anesthesia. Greater volatility of hemodynamics may easily be caused during anesthesia in elderly patients. Furthermore, since anticoagulation therapy is required perioperatively, the risk of postoperative epidural hematomas will rise. It has been reported that in patients received postoperative nerve block anesthesia, the 30-day deep vein thrombosis, postoperative delirium incidence and mortality rates were lower compared with other anesthesia methods [29, 30]. In our patients, the nerve block anesthesia was the first choice. To check for complications of anesthesia, we used a standard neutral position of chest X-ray images (Fig 4).

Potential surgical procedures for femoral neck fracture include total hip arthroplasty, hemiarthroplasty and internal fixation. Geiger et al. [31] demonstrated that in elderly patients, the hemiarthroplasty had more pronounced positive effect than internal fixation. Although there are few reports on bone cement prosthesis leading to a higher occurrence of cardiovascular complications, there have been more studies reporting that the cemented prosthesis had more advantages compared with the uncemented prosthesis [32–34]. Compared with total hip arthroplasty and hemiarthroplasty, hemiarthroplasty has shorter surgical time, less blood loss, and smaller surgical stress and stimulation to patients. Our experience was as follows. For patients older than 65 years old and femoral neck fractures, the hip arthroplasty was the first choice. For patients in good physical condition and with extensive pre-injury activity, total hip arthroplasty should be used. In physically weak patients with poor pre-injury activity of daily living, hemiarthroplasty was a viable option. For intertrochanteric fracture, internal fixation treatment was the preferred selection. In case of hip osteoarthritis, organ failures, or hard to tolerate second surgical procedure, joint arthroplasty could be considered instead.

Limitations of this study were the following. The first limitation is the retrospective nature of the study. Second, patient numbers were low, and there was some patient heterogeneity due to treatment of underlying diseases, physiological disorders, surgical approaches to different fractures, and some variation in anesthesia approaches. These confounders could not be eliminated. Third, the usefulness of the DCO approach to treat the femoral neck fracture in patients with concomitant chronic renal failure were proved only by postoperative complication rates, mortality rates, and Harris scores. Fourth, the long-term effect of the suggested surgical approach requires further verification.

## Conclusions

In summary, using the DCO approach, the surgical treatment for femoral neck fracture in patients complicated with chronic renal failure is an effective and relatively safe treatment option. Increased POSSUM predicted morbidity rate, P-POSSUM predicted mortality rate and serious co-morbidities are associated with adverse outcomes. Following sequential assessment of surgical risk, postoperative mortality rates are significantly reduced. Delaying the surgery for several days, if needed, does not increase the incidence of postoperative adverse events.

## Abbreviations

ASA: American Society of Anesthesiologists
CKD: chronic kidney disease
DCO: damage control orthopedics
ICU: intensive care unit
